# Microencapsulated 3-Dimensional Sensor for the Measurement of Oxygen in Single Isolated Pancreatic Islets

**DOI:** 10.1371/journal.pone.0033070

**Published:** 2012-03-29

**Authors:** Wanyu Chen, Mark Lisowski, Gamal Khalil, Ian R. Sweet, Amy Q. Shen

**Affiliations:** 1 Department of Mechanical Engineering, University of Washington, Seattle, Washington, United States of America; 2 Department of Medicine, University of Washington, Seattle, Washington, United States of America; 3 School of Materials Science and Engineering, Wuhan University of Technology, Wuhan, China; 4 Department of Aeronautics and Astronautics Department, University of Washington, Seattle, Washington, Unites States of America; Boston University, United States of America

## Abstract

**Background:**

Oxygen consumption reflects multiple processes in pancreatic islets including mechanisms contributing to insulin secretion, oxidative stress and viability, providing an important readout in studies of islet function, islet viability and drug testing. Due to the scarcity, heterogeneity, and intrinsic kinetic properties of individual islets, it would be of great benefit to detect oxygen consumption by single islets. We present a novel method we have developed to image oxygen in single islets.

**Methodology/Principal Findings:**

Using a microfluidics system, individual islets and a fluorescent oxygen-sensitive dye were encased within a thin alginate polymer layer. Insulin secretion by the encapsulated islets was normal. Fluorescent signal from the encased dye, detected using a standard inverted fluorescence microscope and digital camera, was stable and proportional to the amount of oxygen in the media. When integrated into a perifusion system, the sensing system detected changes in response to metabolic substrates, mitochondrial poisons, and induced-oscillations. Glucose responses averaged 30.1±7.1% of the response to a metabolic inhibitor (cyanide), increases were observed in all cases (n = 6), and the system was able to resolve changes in oxygen consumption that had a period greater than 0.5 minutes. The sensing system operated similarly from 2–48 hours following encapsulation, and viability and function of the islets were not significantly affected by the encapsulation process.

**Conclusions/Significance:**

An oxygen-dependent dye situated around and within a pancreatic islet encapsulated by a thin layer of alginate was sensitive to changes in oxygen consumption, and was not harmful to the function or viability of islets over the course of two days. The microcapsule-based sensing method is particularly suited to assessing the effects of compounds (dose responses and time courses) and chronic changes occurring over the course of days. The approach should be applicable to other cell types and dyes sensitive to other biologically important molecules.

## Introduction

Oxygen consumption rate (OCR) is a critical parameter to assess as part of investigations of islet physiology and viability. Oxygen is the final acceptor of electrons traversing the electron transport chain (ETC), and in islets oxygen utilization associated with the ETC represents most of the total cellular OCR [1], [2]. Since the rate of the ETC reflects cellular metabolism of nutrients, the ability to generate energy by the cell, and driving forces and signals for oxidative stress and apoptosis. OCR is a window into many aspects of cellular function. Islets in particular increase ATP production as part of the mechanism mediating the secretion of insulin. Accordingly, OCR has been used for islet quality assessment in islet transplantation [3], [4], tests of viability in the optimization of immunoisolation devices [5], [6], studies of beta cells during differentiation from stem cells [7], [8], and mechanistic studies of islet physiology [9], [10], [11].

Due to the importance of OCR for studies of cells of all types, there have been a number of approaches to its measurement. In general, these are broken down into two categories, flow and static systems. Use of flow systems involves measuring the difference between inflow and outflow oxygen content, whereas in the static systems the decrease in oxygen content in a closed chamber is measured over a period of time. Flow systems are able to resolve temporal changes in islet respiration in response to the presence or washout of effector agents. Two static systems that have been used for islet studies include mixed chambers that are equipped with a Clark oxygen electrode [12], [13] and multiwell plates containing an oxygen sensor at the bottom of each well (BD Oxygen Biosensor Systems [14] and Seahorse Bioscience XF Analyzers [15], [16]). A major feature of the plate-based systems is the facility to carry out multiple experiments in parallel.

In general, to be able to measure the decrement in oxygen tension in bulk solution (flow or well-mixed) a relatively large number of islets must be used. A mode of real time analysis of OCR by single islets utilizes oxygen-specific sensors to measure oxygen tension in media surrounding or inside the islet [17], [18]. These measurements are reflective of, but not precisely proportional to, OCR. Nonetheless, the benefits of the single islet analysis are the resolution of intrinsic kinetic responses that are dampened by heterogeneous responses from multiple islets, the small number of islets required to obtain data and ease of characterization of the tissue under interrogation. However, the use of physical sensors has the drawbacks of being invasive, sensitive to the positioning of the sensor within the islet, and incapable of assessing more than one islet at a time. Imaging of single islets using fluorescence-based dyes allows an integrated cellular response in the parameter of interest to be tracked with minimal perturbation to the cell. Commonly imaged parameters in islets include calcium [19], [20], mitochondrial membrane potential [21] and NADH [22]. However, there are no validated oxygen-sensitive fluorescent dyes that can be used to track oxygen tension in or around the islet. We therefore endeavored to develop an imaging method based on the use of platinum porphyrins, oxygen-sensitive dyes that are available in various chemical forms that widely differ in solubility and other chemical properties [23], [24]. In theory, a lipophilic dye could be used which would then situate in the lipid membranes of the islet. However, the dye could only be loaded into islets in the presence of high amounts of DMSO, which would be harmful to the islet. We therefore chose to utilize alginate hydrogels that could be used to encapsulate the islet in the presence of the dye, thereby trapping the dye in the extracellular fluid around and within the islet.

Encapsulation has been tested as a way to provide an immunoprotective barrier when transplanting islets into diabetic patients [25]. Materials used in the encapsulation process such as alginate are porous, permitting the exchange of nutrients and cellular waste products to the surrounding solutions. Collectively, studies have shown that islet viability is maintained during and after the encapsulation process both in vitro and in vivo [26]. Nonetheless, using traditional cell encapsulation procedures it has been difficult to make spherical shaped microcapsules in the optimal size range for islets (100–200 µm) [27].

Recently, microfluidic techniques have advanced cell encapsulation by using hydrogels or other biopolymers as the microcapsules [28]. This approach preserves cell viability and generates a uniform set of monodisperse microencapsulated cells necessary to obtain reliable individual cell behavior from a population of cells [29]. Tan and co-workers [30] used microfluidic devices to generate microcapsules where the polymerization of the microcapsule surrounding Jurkat cells occurred following a T-junction that mixed the cells and the solutions catalyzing the polymerization. In this paper, we used such a T-junction to encapsulate single isolated pancreatic islets and a water-soluble, oxygen-sensitive, fluorescent dye inside a 180- µm sized alginate microcapsule. The dye situates itself during the encapsulation process in the extracellular space between the islet cells and the alginate layer, and acts as a 3-dimensional real time oxygen sensor. The alginate microcapsule-based sensor was stable, sensitive to small changes in oxygen tension, and responded to various effectors of mitochondrial metabolism in real time. Our approach can be used to screen the effects of compounds on OCR in single islets, and also could be adapted for use with other fluorescent dyes sensitive to many compounds of biological importance to islets and other cells.

## Materials and Methods

### Chemicals

Krebs-Ringer bicarbonate solution (KRB), used for all perifusion analysis, contained 98.5 mM NaCl, 4.9 mM KCl, 1.2 mM potassium phosphate, 1.2 mM magnesium sulfate, 25.9 mM sodium bicarbonate, 2.6 mM CaCl_2_, varying amounts of glucose (all from Sigma Aldrich, St. Louis, MO), and 20 mM HEPES (Research Organics, Cleveland, OH). The agents used in perifusion experiments were potassium cyanide (KCN), carbonyl cyanide 4-(trifluoromethoxy)phenylhydrazone (FCCP), and nimodipine (Sigma-Aldrich). Additional agents used in the encapsulation procedure were soybean oil (shear viscosity = 50 mPa*s, produced by Cibaria, Riverside, California), Sterile sodium alginate (endotoxins<100 EU/g) (Novamatrix, Oslo, Norway), and the detergent Span 80 (Sigma-Aldrich). The oxygen-sensitive dye, Pt(II) meso-Tetra(N-Methyl-4-Pyridyl)Porphine Tetrachloride, was purchased from Frontier Scientific (Logan, Utah).

### Rat islet isolation and culture

Islets were harvested from Sprague-Dawley rats (≈250 g, Charles River) anesthetized by intraperitoneal injection of sodium pentobarbital (35 mg/230 g rat). All procedures were approved by the University of Washington Institutional Animal Care and Use Committee. Islets were prepared and purified as previously described [1], and then cultured at 37°C in RPMI Media 1640 (Gibco, Grand Island, NY) supplemented with 10% heat-inactivated fetal bovine serum (Atlanta Biologicals, Lawrenceville, GA) for 18 hours prior to encapsulation.

### Encapsulation/dyeing of islets

**Figure 1:**
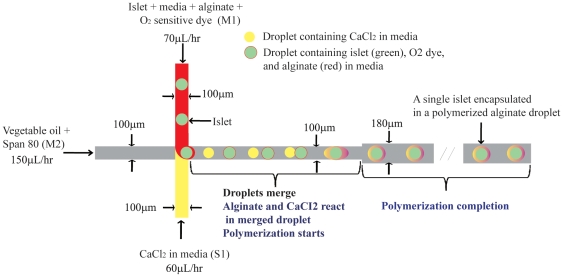
Schematic of microfluidics system used to encapsulate islets. Islets were encapsulated in polymerized alginate using a microfluidic system with three rectangular inflow channels (100 µm wide) and a single outflow channel (180 µm wide). Each microchannel has a depth of 180 µm. A mixed solution of soybean oil and Span 80 (0.05%), acting as the carrier phase, was pumped through the center microchannel at 150 mL/hr using a syringe pump. Culture media containing 0.05 M CaCl_2_ (lower microchannel) or islets/alginate (0.2 M)/oxygen-sensitive dye (20 µM) (upper microchannel) was pumped at 60 or 70 mL/hr respectively. At the intersection of the microchannels, the aqueous phases enter the faster flowing oil phase in alternating fashion as discrete droplets. Once in the main channel, the less viscous and slightly faster CaCl_2_ droplet collides and merges with the more viscous alginate droplet. During the 3-min transit through the outflow channel, calcium causes the alginate surrounding the islet to polymerize thereby encasing both islet and oxygen-sensitive dye by a thin alginate layer.

As shown in Fig. 1, islets were encapsulated in alginate using a microfluidic system with three inflow channels (100 µm wide) and a single outflow channel (180 µm wide), fabricated using soft lithography [31] (Fig. S1 and Fig. S2). A detailed description of the construction of the microfluidic device, the encapsulation process using the microfluidics device and the real time assessment of islet oxygen is provided in the Appendix S1. The cross section of each microchannel was rectangular with a depth of 180 µm. A mixed solution of vegetable oil and Span 80 (1% volume ratio), acting as the carrier phase, was pumped through the center microchannel at 150 µL/hr. Culture media containing 45 mM CaCl_2_ was pumped through the lower microchannel at 60 µL/hr and another aqueous mixture of culture media containing islets, alginate (1% wt ratio), and oxygen-sensitive dye (20 µM) was pumped through the upper microchannel at 70 µL/hr (Fig. 1).

At the intersection of the microchannels, the aqueous phases enter the faster flowing oil phase in alternating fashion as discrete droplets in the face of competition of viscous and capillary forces. Once in the main channel, the less viscous and faster calcium droplet collides and merges with the more viscous alginate droplet containing the oxygen-sensitive dye and islet. Polymerization occurs in the outflow channel when the alginate comes into contact with Ca^2+^ thereby trapping the dye along with the islet. Encapsulated/dyed islets were collected from the outflow into a vial of culture media, and centrifuged at 1500 g for 3 minutes to separate the oil from the media and islets. Encapsulated islets were transferred to a petri dish with fresh culture media, and incubated in a CO_2_/37 degree incubator for 2–48 hours prior to experimentation.

### Imaging of oxygen levels at the plasma membrane

**Figure 2:**
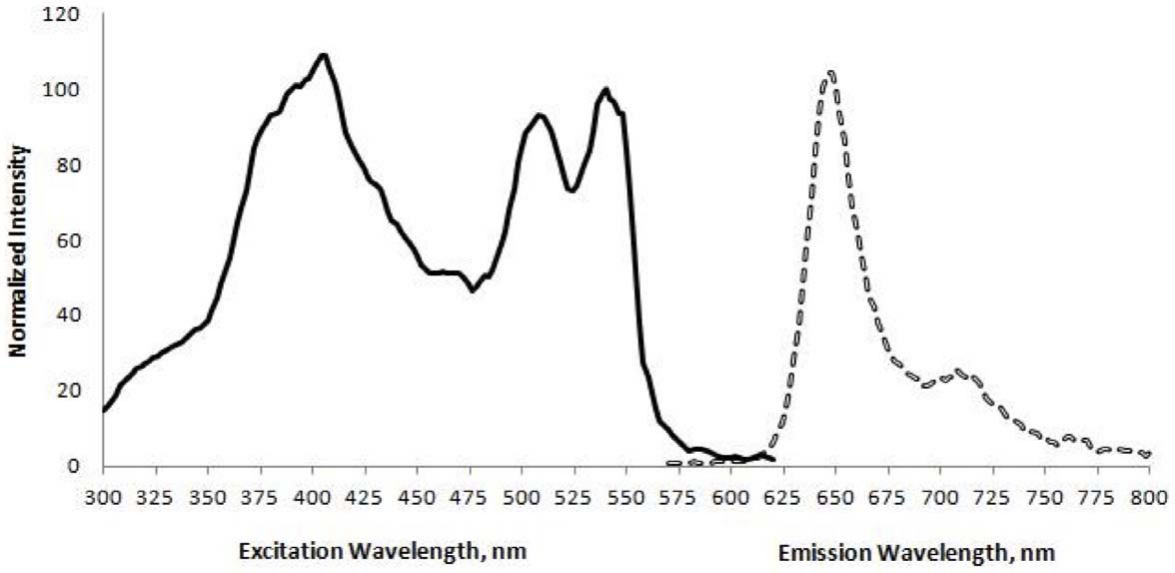
Excitation and emission spectra of the oxygen-sensitive dye Pt(II) meso-Tetra(N-Methyl-4-Pyridyl)Porphine Tetrachloride. The excitation spectrum represents emission at 650 nm while varying the excitation wavelength from 300 to 625 and was normalized to the intensity from excitation at 540 nm. The emission spectrum was obtained with the excitation wavelength set to 540 nm and was normalized to the emission intensity at 650 nm.

Encapsulated islets were pipetted into a temperature-controlled, 250-µl perifusion dish (Bioptechs, Butler, PA), which was then sealed and mounted on to the stage of a Nikon Eclipse TE-200 inverted microscope. KRB was pumped through the dish at a flow rate of 150 µL/min using a Masterflex L/S peristaltic pump (Cole-Parmer, Vernon Hills, IL). To control gas tension in the perifusate, KRB flowed through an artificial lung consisting of 24 cm of gas-permeable silastic tubing (1.57 mm i.d., 2.41 mm o.d.) loosely coiled in a 125 mL glass bottle (continuously supplied with 1 ml/min of 21% oxygen and 5% CO_2_) prior to entering the perifusion dish. The perifusion system we have developed is similar to many standard systems [45]. Using a digital camera (Photometrics Cool Snap EZ, Tucson, AZ), oxygen was imaged in encapsulated islets by detecting emission between 580–640 nm (at 400× magnification) in response to excitation at 541–551 nm. The full excitation and emission spectra of the oxygen dye are shown in Fig. 2, generated using a Synergy 4 Multi-Mode MicroPlate Readers (BioTek, Winooski, VT). More detailed description can be found in the Appendix S1.

### Assessment of insulin secretion

Insulin secretion rate was determined statically with multiple conditions as previously described [32]. Briefly, encapsulated and unencapsulated islets were handpicked in parallel into a Petri dish containing KRB, 0.1% BSA and 3 mM glucose and incubated at 37°C/5% CO_2_ for 60 min. Subsequently, islets were placed into wells of 96-well plates (10 islets/well) containing indicated amounts of glucose, and incubated for 60 more min. At the end of this period, supernatant was assayed for insulin using an RIA kit (Linco Research Inc., St. Charles, MO).

## Results

### Morphology, dye distribution, and secretory function of encapsulated islets

**Figure 3:**
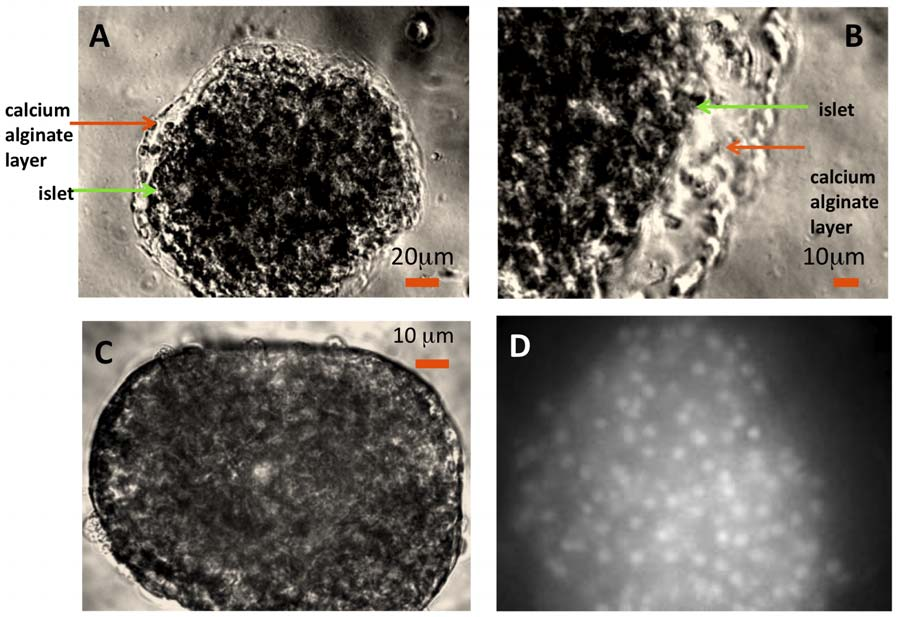
Images of encapsulated islets illustrating the polymerized alginate layer and dye fluorescence. Islets were placed in the perifusion chamber and brightfield images were taken with a digital camera as described in the Methods section. Images of single islets (A. diameter = 140 µm; B. diameter = 95 µm) coated with transparent calcium alginate. C. Image of unencapsulated islet. D. Fluorescence image of an encapsulated/dyed islet.

The microcapsule size of the encapsulated islet is determined by the width of the outflow microchannel (180 µm). Thus, the thickness of the alginate layer surrounding the islet was the difference between the size of the islet and 180 µm. The alginate layer is nearly transparent, but was visible in images obtained with brightfield illumination (Fig. 3A and B); no layer was observed in the unencapsulated islet (Fig. 3C). Typically, the thickness of the alginate layer was uniform. For a 145-µm islet the alginate layer was about 15 µm thick (Fig. 3A) and for a 90-µm islet the layer was about 45 µm thick (Fig. 3B). When the oxygen-sensitive dye was included in the encapsulation solution, the resulting encapsulated/dyed islet fluoresced in all regions of the islet, most brightly in areas around cells in the islet (Fig. 3D).

**Figure 4:**
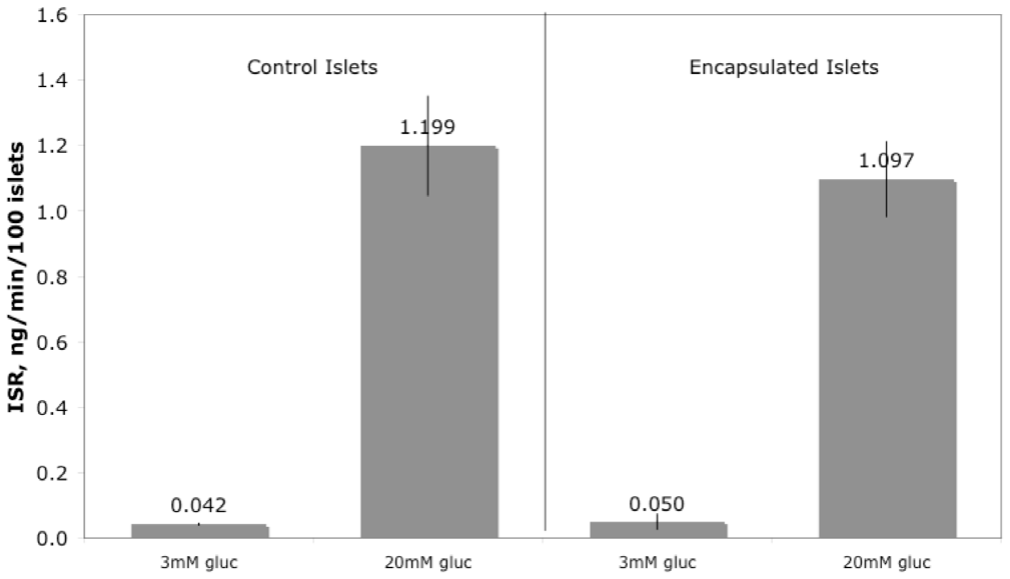
Insulin secretion by encapsulated and unencapsulated islets. After pre-incubation in KRB containing 3 mM glucose, islets were incubated in 96-well plates in the presence of 3 or 20 mM glucose for 60 min. Supernatant was collected and later assayed for insulin.

The flow rates were adjusted so that coalescence of calcium-containing and islet-containing droplets would occur. In addition, the flow rates were kept low in order to avoid excessive shear stress. The maximal shear stress acting on the islets within the microfluidic chamber was estimated to be around 2.3 Pa (calculated as the viscosity of the soy oil×islet velocity/width of channel = (0.05 Pa*s×4.6 mm/s)/0.1 mm), which would occur at the outflow tube after the T-junction. This is in the same range as shear stress experienced by endothelial cells in vivo [33]. In order to gauge the functionality of the encapsulated islets, their ability to secrete insulin was compared to that of unencapsulated islets. Islets, cultured for 18 hours following encapsulation, were handpicked into Petri dishes containing KRB buffer with 3 mM glucose. After 60 min, islets were transferred into 96-well plates (10 islets/well) containing KRB buffer with 3 or 20 mM glucose. Buffer was removed from the wells after 60 min, and insulin in the buffer was subsequently measured using an RIA kit. Insulin secretion rate was increased in response to 20 mM glucose by encapsulated islets similarly to unencapsulated (1.2±0.16 vs. 1.1±0.12 ng/min/100 islets, Fig. 4). Thus, the process of encapsulation and the alginate layer does not impede the secretion of insulin.

### Effect of flow rate on oxygen in a single islet

**Figure 5:**
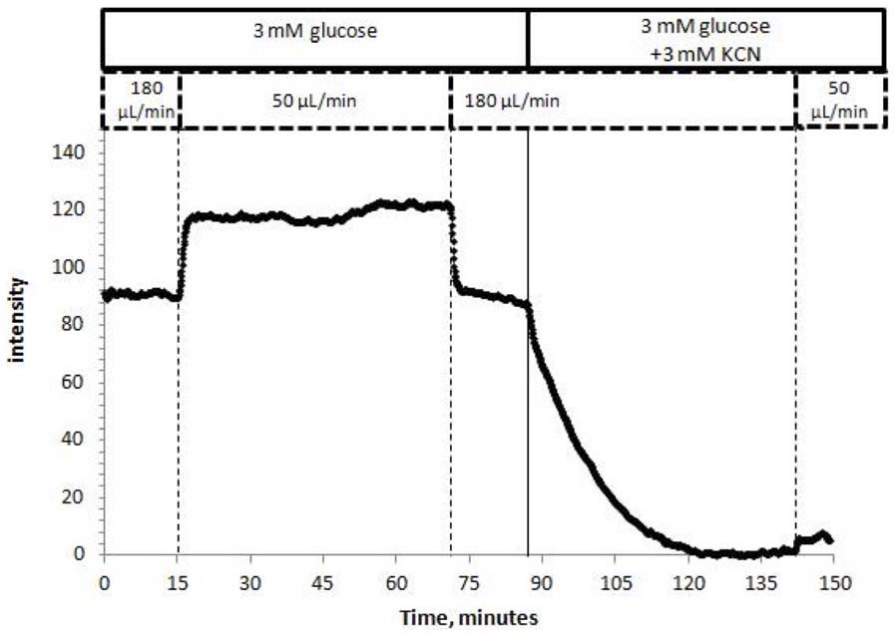
Effect of perifusion flow rate on islet oxygen. Encapsulated islets were perifused at 180 µL/min in KRB containing 3 mM glucose. At time = 15 minutes, the flow rate was changed to 50 µL/min, and at time = 70 it was decreased back to 180 µL/min. This protocol was repeated after suppressing respiration by 3 mM KCN.

The oxygen tension around and in the islet is a balance between oxygen usage by the islets and its repletion via diffusion and flow. Decreasing flow from 180 to 50 µL/min resulted in an immediate decrease in oxygen tension, which was reversed after returning to the initial flow (Fig. 5). Changing flow rate in the presence of a respiratory inhibitor (KCN) had no effect on oxygen tension, demonstrating that respiration was the major determinant in changes of detected oxygen. Therefore, KCN was added at the end of each experiment and the steady state value of oxygen tension in its presence was set to zero. Since islet viability is very sensitive to inadequate supply of oxygen, subsequent experiments were conducted at a flow rate of 180 µL/min or greater.

### Calibration of oxygen sensor signal as a function of dissolved oxygen in buffer

**Figure 6:**
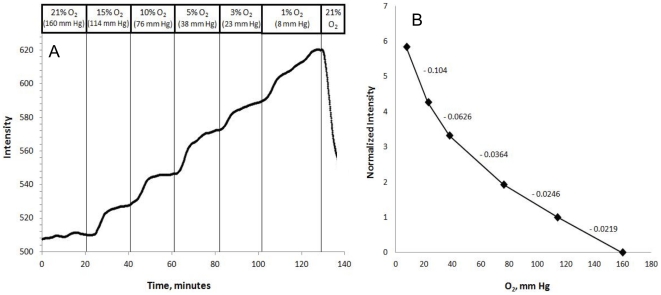
A. Effect of oxygen tension in the perifusate on dye in islet. Rat islets were encapsulated with the oxygen-sensitive dye and then loaded into the perifusion system. Fluorescence from the dye was measured after islet respiration was suppressed by 3 mM KCN. Oxygen tension in the perifusate was varied at the indicated time by supplying the indicated gas mixtures to the system's artificial lung every 20 min as shown. **B. Steady-state intensity values for each oxygen level**. Intensity was averaged over the final 5 minutes of each period, normalized to the change in intensity observed in response to decreasing oxygen tension from 142 to 101.5 mm Hg, and plotted as a function of oxygen tension in the perifusate. Numbers for each segment of the curve are the slopes as intensity/mm Hg.

To calibrate the dye signal, fluorescent emission from encapsulated/dyed islets was measured at different levels of dissolved oxygen tension after islet respiration was suppressed by 3 mM KCN. Oxygen tension was varied by hooking up various tanks of gas containing 5% CO_2_, and either 21, 15, 10, 5, 3, or 1% O_2_ with balance N_2_ to the gas equilibration system. The oxygen signal reached steady state about 5 min after the change in oxygen (Fig. 6A), although much of this delay was due to the time it took oxygen in the gassing system to affect a change in the dissolved oxygen content of the buffer. Blowing gas directly on the sensor generated a change in signal that reached steady state in a few seconds. Fluorescence intensity increased with decreasing oxygen tension, consistent with the known quenching properties of oxygen to the fluorescent emission (Fig. 6A). In order to construct a calibration curve, steady state values of fluorescence were calculated by averaging the final 5 minutes of intensities for each oxygen tension. These were plotted as a function of oxygen tension (Fig. 6B). The shape of this curve was similar to calibration curves obtained using a fluorometer [24].

### Response of islet oxygen to glucose, mitochondrial uncoupling, and inhibition of respiration

**Figure 7:**
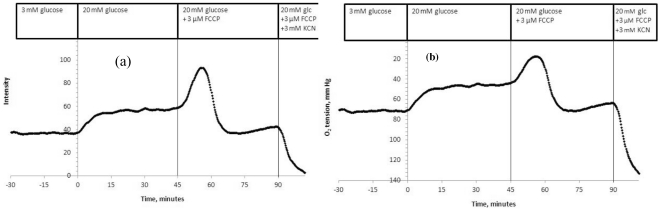
A. The effect of substrate, mitochondrial uncoupling and inhibition of respiration on dye intensity in encapsulated rat islets. Encapsulated rat islets were perifused in the presence of 3 mM glucose for 60 min (only the final 30 min is shown). Glucose was increased to 20 mM at time 0. Respiration was subsequently uncoupled with 3 µM FCCP for 45 min and then inhibited with 3 mM KCN. Intensity was normalized as described in Fig. 6B. **B. Intensity data converted to oxygen tension.** Using the oxygen calibration curve shown in Fig. 3, intensity was converted to corresponding oxygen tension. Glucose (20 mM) decreased oxygen tension by 24.7 mm Hg and FCCP caused a further transient decrease of 28.3 mm Hg.

The method was evaluated by measuring the response of encapsulated islet oxygen to agents that we have previously characterized with respect to their effect on OCR. After approximately 60 min at 3 mM glucose, oxygen tension in the extracellular fluid surrounding the islet was very stable (Fig. 7A). Increasing glucose from 3 mM to 20 mM resulted in an increase in fluorescence (consistent with a decrease in islet oxygen tension and an increase in OCR). The intensity further increased by mitochondrial uncoupling (using FCCP), although it subsequently decreased, presumably due to the toxic effect of high levels of FCCP. Exposure of islets to KCN, an inhibitor of mitochondrial respiration, rapidly decreased islet oxygen tension. This entire protocol was repeated 6 times with islets that had been cultured for either 2, 18 or 40 hours. All 6 islets responded similarly to these three agents (glucose responses averaged 30.1±7.1% of total respiration rate).

Fluorescent images of the encapsulated/dyed islets showed that dye penetrated into the islet and gave the strongest signal from regions near cells (Fig. 3D). However, it was not clear whether the dye was entering cells, or the signal was just stronger around the cells due to gradients of oxygen tension which would arise from consumption. To determine whether the dye was incorporated into the islet cells, rather than in the extracellular compartment of the encapsulated islet, unencapsulated islets were exposed to the oxygen sensitive dye (20 µM) for 60 min and fluorescence of the oxygen dye in the islets was imaged. A fluorescent signal was detectable, but it did not respond to glucose or KCN. Thus, the signal from the oxygen-sensitive dye, a highly water-soluble compound that was not expected to be permeable to the cell membrane, appeared to emanate from a three dimensional region within the alginate layer surrounding the plasma membrane of the islet cells.

The steady-state values of fluorescent intensity as a function of oxygen in the buffer shown in Fig. 6B were used to convert the fluorescent emission signal to oxygen tension in mm Hg. At 3 and 20 mM glucose, oxygen tension ranged between 75 and 45 mm Hg (Fig. 7B), values that were not far from what has been reported to be physiologic [34]. After mitochondrial uncoupling induced by the addition of FCCP, islet oxygen dropped to near-anoxic condition.

### Resolution of oscillatory behavior in islets

**Figure 8:**
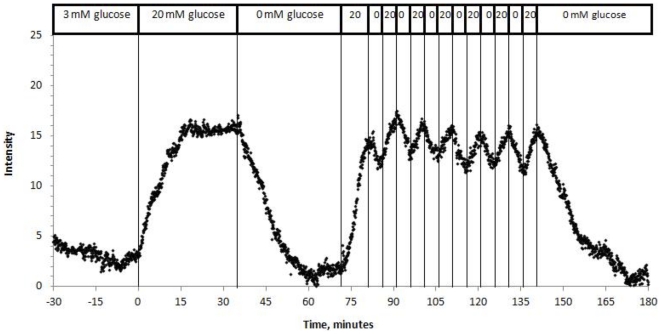
Resolution of induced oscillations in rat islet respiration. Encapsulated rat islets were perifused in the presence of 3 mM glucose for 60 min. Glucose was increased to 20 mM at time 0 and then decreased to 0 mM after 35 min. Subsequently, glucose was increased to 20 mM for ten min, and then glucose was changed between 20 mM and 0 mM every 5 min for one hour.

To evaluate the microcapsule method's ability to detect periodic changes in OCR, glucose in the inflow was alternatively increased and decreased at 5-min intervals (Fig. 8). Oscillations in islet oxygen closely tracked the temporal changes in glucose with amplitude of about 27% of the steady state change in glucose-stimulated oxygen tension. The amplitude was reduced because it took about 4 minutes to reach inflow glucose concentrations below 2 mM. A pulse train of 6 glucose pulses generated very consistent waveforms of similar magnitudes. Changes in oxygen with a periodicity of 5 minutes were easily resolvable. There were however, occasional rapid transient spikes on the order of 20 seconds or less that were indistinguishable from noise (i.e. occasional spikes were also seen in oxygen profiles in the absence of islets). Accordingly, data was smoothed for spikes that occurred with a peak width of less than 20 seconds. Thus, a limitation of the temporal resolution is estimated to be about 30 seconds. When rat islet oxygen was measured for 40–60 minutes at 20 mM glucose (without glucose pulsing), no periodicity was seen in the oxygen profiles.

## Discussion

### Features of microfluidics-generated microcapsules for sensing islet oxygen

Although a number of microsensors have been used for measuring oxygen tension in islets [18], [35], the use of microencapsulated oxygen-sensitive dye offers several advantages. The first is that optical sensing of oxygen is only minimally invasive, allowing responses to changes in inflow composition to be assessed without perturbations to the islets. Second, the optical signal reflecting islet oxygen is an integration of the oxygen tension from within and around the islet cells, ensuring that the method yields data that is representative of the whole islet. Third, the droplet method measures oxygen right at the plasma membrane that includes intra-islet spaces. Finally, the method is particularly useful when it is desired to measure changes in OCR, but only very small numbers of islets are available. These capabilities make the method useful for general studies on islet physiology, testing of drugs on islet metabolism, assessment of islet cells produced by differentiation of stem cells, and assessment of islet quality for islet transplantation.

### Validation of islet oxygen method

Assessing the validity of the encapsulated sensor method was based on both insulin secretion and islet oxygen measurements. Twenty-four hours after encapsulating islets in the presence of oxygen-sensitive dye, insulin secretion rate was not different from insulin secretion from unencapsulated islets. Thus it appears that the alginate layer does not significantly impede exchange of glucose and insulin across the islet cell membranes. The interpretation of islet oxygen results is less direct, and relied on comparison to OCR measurements of a population of islets in response to glucose, a mitochondrial uncoupling agent and inhibitor of cellular respiration (KCN). Consistent with OCR measurements, cellular respiration was responsible for the vast majority of the islet oxygen signal, since in the presence of KCN, islet oxygen was no longer sensitive to flow rate changes. Regarding the accuracy of the encapsulated sensor method to quantify changes in islet oxygen in response to agents, it is difficult to ascertain whether there are systematic errors. However, the responses to the three agents (as well as flow rate) indicate that the observed change in islet oxygen reflects changes in OCR by metabolically active islets. Moreover, responses to these three agents were observed in all six of the islet perifusions run with this protocol, illustrating the reliability of the measurement approach.

### Selection of oxygen tension under which to conduct islet experiments

The vast majority of islet studies are performed using media that is equilibrated with either 21% oxygen, or in order to prevent any effects of oxygen limitation, with 95% oxygen. However, physiological oxygen tensions in the islet are thought to range between 40 and 25 mm Hg. The perifusion system used permits the adjustment of islet oxygen either by choice of the oxygen content of the gas canister that is supplying the artificial lung, or by altering the flow rate of the perifusion. When the inflow perifusate was equilibrated with 21% oxygen, the islet oxygen ranged between 72 and 43 mm Hg, a little above what is physiologic. However, these values are integrated signals from the entire islet, and it is likely that oxygen at the core of the islet is much lower than this. It may be that conducting the experiment using oxygen tanks with a higher content of oxygen would give more robust results. It may also be useful to investigate the effects of compounds on changes in islet oxygen at different oxygen tensions.

### Temporal resolution of islet oxygen detection

Oscillations in islet metabolism have been postulated to drive oscillations in calcium observed in mouse islets and oscillations in secretory function [36], [37], [38]. Therefore the availability of a method that can resolve oscillations in OCR would be very useful to characterize the temporal behavior and the factors that may regulate oxygen kinetics. The method we developed was only partially successful in this regard. The method was able to resolve small changes (10%) in islet oxygen with a period of 5 minutes induced by changes in glucose concentration. However, random spikes that occurred both in the absence and presence of metabolically active islets limited the ability to resolve oscillatory kinetics with periods of less than 30 sec. We expect that the rapid spiking we observed could be eliminated by the use of a camera capable of measuring lifetime of fluorescence decay, a parameter that is insensitive to fluctuations in power and quenching of excitation light. At this point, we can say that the rat islet oxygen data that was obtained at high glucose for 40 minutes did not reveal oscillations, but we cannot say whether OCR was oscillating rapidly with fast periods.

### Cell encapsulation using microfluidic devices

Using microfluidics technology, we have presented a method for islet microencapsulation that appears to trap a fluorescent dye and support islet viability over a period of 2 days or more. The microfluid system we have used is particularly effective at generating and manipulating uniform micron-sized microcapsules. There are a number of options that could be considered with respect to optimizing the method, most importantly the choice of polymer layer. Alginate is widely used for cell immobilization and encapsulation because it is biocompatible and can be polymerized by simple gelation with divalent cations such as calcium. Since calcium can induce apoptosis in islets, however, it may be better to use zinc [39] or barium as the cross linker. ARPE-19 cells have been successfully encapsulated into Ca^2+^–Ba^2+^ cross-linked alginate microcapsules and these cells remained viability for at least 110 days in the microcapsules [40]. It has been suggested that polyethylene glycol (PEG) may stabilize alginate and it has been used to form covalently linked barium-alginate-PEG microcapsules around rat and human pancreatic islets [26]. Glucose stimulated insulin secretion was still robust after 5 days in culture. Other choices of polymers that have been used successfully for encapsulation of cells include hyaluronic acid hydrogel beads [41], poly(ethylene glycol) hydrogels [42], and alginate-poly-L-lysine-alginate [43]. These may improve the resolution and precision of the oxygen measurements or the functionality of the encapsulated islets. It is also possible to incorporate enzymes or other molecules to support growth and viability, such as was done with superoxide dismutase to reduce the cell's exposure to superoxide mediated damage [42].

### Future Applications

The microcapsule method that we have presented has utility as a general method for examining the effects of agents on OCR. Although a microsensor previously applied to islets appears to have greater temporal resolution than our method [35], there are several unique features of the approach that could be the basis for additional methodologies over and above what was illustrated in this manuscript. For instance, the stability and inert nature of the sensor dye over the course of 2 days make possible long term experiments with that would involve continuous measurement of oxygen over days. In addition, there are a number of dyes sensitive to parameters involved in the regulation of insulin secretion in the islet, including calcium, NADH and mitochondrial membrane potential. These dyes could be dual loaded with the oxygen-sensitive dye, and the simultaneous measurement of both would allow for kinetic relationships between these interconnected variables to be characterized.

Our microcapsule sensor method, although complex relative to the oxygen consumption methods developed by BD Biosciences (Oxygen BioSensor System Microplate [14]) and Seahorse Bioscience (Seahorse Extracellular Flux (XF) Analyzer [44]) may also be adaptable to a high-throughput screening format. This would be accomplished by placing encapsulating islets into 96-well plates and measuring fluorescence in the wells using standard fluorometric plate readers. Finally, since many dyes sensitive to intracellular molecules or processes are cell-impermeable, the method should be able to encapsulate additional dyes. The interrogation of the region contiguous with the extracellular side of the plasma membrane yields complementary information to intracellular sensors. For instance, the detection of an extracellular dye sensitive to reactive oxygen species would reflect the total net cellular production if the dye was encased around the islet, whereas intracellular detection represents the balance between production, transport and quenching occuring between various organelles and enzymes. Overall, the use of microfluidics to encapsulate fluorescent dyes along with islets opens up a whole spectrum of measurement modalites. In addition, we expect that the method will be transferrable to the study of OCR by other cell types or cell clusters.

## Supporting Information

Appendix S1 **Detailed description of the construction of the microfluidic device, the encapsulation process using the microfluidics device and the real time assessment of islet oxygen.** See text in Appendix S1 for details.
(DOCX)

Figure S1 **Steps involved in microdevice fabrication.** See text in Appendix S1 for details.
(TIF)

Figure S2 **A photo image of the microdevice used for encapsulation of islets.** See text in Appendix S1 for details.
(TIF)
