# Supplementary material for: Microencapsulated 3-Dimensional Sensor for the Measurement of Oxygen in Single Isolated Pancreatic Islets
Source: PLoS One. 2012 Mar 29;7(3):e33070. doi: 10.1371/journal.pone.0033070 (PMC3315556; doi:10.1371/journal.pone.0033070)
Supplement: Appendix S1 — Detailed description of the construction of the microfluidic device, the encapsulation process using the microfluidics device and the real time assessment of islet oxygen. See text in Appendix S1 for details. (DOCX) [file pone.0033070.s001.docx]

## SUPPORTING INFORMATION: DETAILED METHOD DESCRIPTION

The steps involved in the method to encapsulate, dye, and image islet oxygen tension are relatively complicated, so we have provided a detailed, step by step protocol of what was done to generate the data in the manuscript. We first describe the methodology used in fabricating the microfluidics device needed to carry out the encapsulation of the islet. This is followed by the protocol for encapsulating and dyeing the islet with the oxygen-sensitive dye. Finally, we describe the procedures for using the perifusion/imaging systems to measure change in oxygen tension in real time.

**1. Photolithography: preparation of the silicon mold.**

The first step in constructing the microfluidics device was to generate a mold (or master) for the microchannels. In order to form a template for the microchannel design, a viscous polymer (SU-8, MicroChem, Newton, MA) was uniformly coated onto a silicon wafer at a thickness of 180 microns, and baked to harden the SU-8 (called a photoresist) (Fig. A1, step 1). The wafer was then covered with an overlay that masked the negative feature of the channel pattern (Fig. A1, step 2). The wafer and mask were then exposed to UV light, which cross-linked and solidified the exposed areas of the photo-resist. The unexposed areas were rinsed off and a ridged pattern in the shape of the mask was left on the silicon wafer (Fig. A1, step 3).

**2. Soft lithography: production of** [**polydimethylsiloxane**](http://en.wikipedia.org/wiki/Polydimethylsiloxane)  **(PDMS) microfluidic device from mold.**

The silicon wafer was used to make multiple microchannel devices (in a process known as soft lithography) by first pouring PDMS polymer over the wafer (Fig. A1, step 4). The PDMS covered wafer was then placed in a vacuum chamber to remove any air bubbles before curing it in a convection oven. The patterned region of the hardened PDMS slab was cut out and removed from the master (Fig. A1, step 5), and inflow and outflow ports were punched in the channels. In order to enhance bonding the PDMS to a glass cover, the PDMS slab was exposed to a plasma gas, which rearranges the terminal hydroxyl groups on the surface of the PDMS. The PDMS slab was then placed onto a glass microscope slide that was previously dried in an oven (Fig. A1, step 6). After 24 hours, a permanent covalent bond formed between the two surfaces creating a sealed fluid network. The microfluidics device used in the encapsulation of the islets is shown in Fig. A2. Fluids were pumped in to and out of the network through small bore (poly) ethylene-oxide tubing that was inserted into the outflow port.

3. **Encapsulation of individual islets.**

To encapsulate islets with the microfluidics device, the following solutions were prepared fresh.

*Solution 1. Islet culture media containing 200 μM of the oxygen-sensitive dye Pt(II) meso-Tetra(N-Methyl-4-Pyridyl)Porphine Tetrachloride (Frontier Scientific, Logan, UT) and 1wt% sodium alginate (Novamatrix, Oslo, Norway).* This solution was prepared by dissolving 2 mg of dye into 5 ml of 2x concentrated RPMI 1640 (containing 20%FBS), and subsequently adding 2wt% sodium alginate at a 1:1 volume ratio.

*Solution 2. Carrier oil phase.* Span 80 (*Sigma-Aldrich, St. Louis, MO*) was added to vegetable oil (*Cibaria, Riverside, CA*) at a 1% volume ratio.

*Solution 3.* *Calcium-containing media.* RPMI1640 (10% FBS) was supplemented with 45 mM CaCl_2_.

The procedure was carried out by pipetting 100 islets into a microcentrifuge tube containing 0.5 mL of solution 1 and the contents were sucked into 30 cm of fluorinated ethylene propylene (FEP) tubing (ID = 0.76 mm, IDEX health & Science, Oak Harbor, WA) using a 1 mL plastic syringe (BD, Franklin Lakes, NJ). The syringe was then placed into a syringe pump (Harvard Apparatus, Holliston, MA) and the tubing was inserted into the elastic PDMS microchannel of the microfluidics device (see Fig. A2). Similarly, solution 2 (~0.7mL) and solution 3 (~0.7mL) were loaded into 1 mL syringes with tubing separately, and these syringes were placed in 2 additional syringe pumps.  The three solutions were pumped at flow rates of 150, 60 and 70 μL/hr for typically 90-120 minutes. Encapsulated islets flowed out of the microfluidics device through 10 cm of FEB tubing into a 10 mL vial of islet culture media, centrifuged at 1500 g for 3 minutes to separate the oil from the media and islets.

**4. OCR Imaging procedure for dyed islets.**

*Perifusion System Set-up.* The perifusion system we have developed is similar to many standard systems [45]. However, the use of CO_2_-based buffers demands that care is taken to avoid bubble formation. To avoid creating a driving force for de-gassing, the perifusate (KRB containing the indicated agents) was placed upright in a water bath heated to 40°C to create buffer that is slightly sub-saturated with respect to oxygen and CO2. From conical tubes, the buffer was pumped through 1 mm i.d. PEEK tubing and a 6-port selection valve (Upchurch Scientific, Oak Harbor, WA) using a Masterflex L/S peristaltic pump (Cole-Parmer, Court Vernon Hills, IL) loaded with 0.25 mm i.d. Masterflex PVC tubing. The outflow of the pump tubing was attached to a 10 cm section of PEEK tubing that extended into the heated (37°C) environmental chamber (Nikon) surrounding the imaging microscope and fed into the gas equilibrator (25 cm of 1.57 mm i.d./2.41 mm o.d. gas permeable silastic tubing (Dow Corning Corporation, Midland, MI) loosely coiled inside of a 125 mL Erlenmeyer flask). The flask was continuously supplied with 1 ml/min of 21% oxygen and 5% CO_2_. Situated between the outflow of the lung and the temperature controlled cell chamber (FCS2, Bioptechs Inc., Butler, PA), was a bubble-trap made from borosilicate glass tubing (3.5 cm length, 3 mm i.d.) containing two porous plugs (5 mm long, made by coring polyethylene sheets obtained from Small Parts Inc. (Miami Lakes, FL)). The outflow of the cell chamber was collected using a fraction collector positioned outside of the heated environmental chamber.

*Loading of Encapsulated/Dyed Islets.* Once the tubing system was set up and the temperature of the water bath and environmental chamber were equilibrated to 40 and 37 °C, respectively, the fluidics system was primed by pumping KRB at 150 µL/min, typically for 2 hours, ensuring that any air bubbles in the tubing and chamber were carried out with the flow. Once the system was primed, the lid of the cell chamber was removed, 5-20 encapsulated/dyed islets were pipetted into the center of the chamber, the chamber lid was resealed, and the whole dish was fastened onto the microscope stage. To ensure complete equilibration of the islet with its new environment and to allow for the baseline to stabilize, the perifusion system was allowed to run for 60-90 minutes before the test protocol was started.

*Imaging of islet fluorescence.* The oxygen dye’s fluorescence was imaged by excitation at 541-551 nm with an Xenon lamp and measuring the emission from 580-640 nm through a 40x Superfluor objective (Nikon Eclipse TE-200 inverted microscope) with a Photometrics Cool Snap EZ digital camera (Tucson, AZ). (Alternatively, a filter centered at 650 nm should increase the signal acquired.) Acquisition times were 50 msec and sampling frequency was either every 5 or 15 sec, conditions that did not produce photobleaching or degradation of the dye signal. Since the dye is temperature sensitive, the use of the temperature controlled cell chamber was very important.

*Conducting the experimental protocol.* During the course of the experiment, the composition of the KRB solution flowing over the islets was changed using the selection valve. At the end of the experiment, 3 mM KCN was spiked into the conical of the solution that was actively flowing, so that the outflow oxygen tension became virtually the same as the inflow. In order to calibrate the fluorescent signal, the percentage of oxygen in the gas mixture supplied to the artificial lung was varied (typically 21, 10, 5 and 3% O2 were used).
