# Supplementary figures and images for: Microencapsulated 3-Dimensional Sensor for the Measurement of Oxygen in Single Isolated Pancreatic Islets
Source: PLoS One. 2012 Mar 29;7(3):e33070. doi: 10.1371/journal.pone.0033070 (PMC3315556; doi:10.1371/journal.pone.0033070)

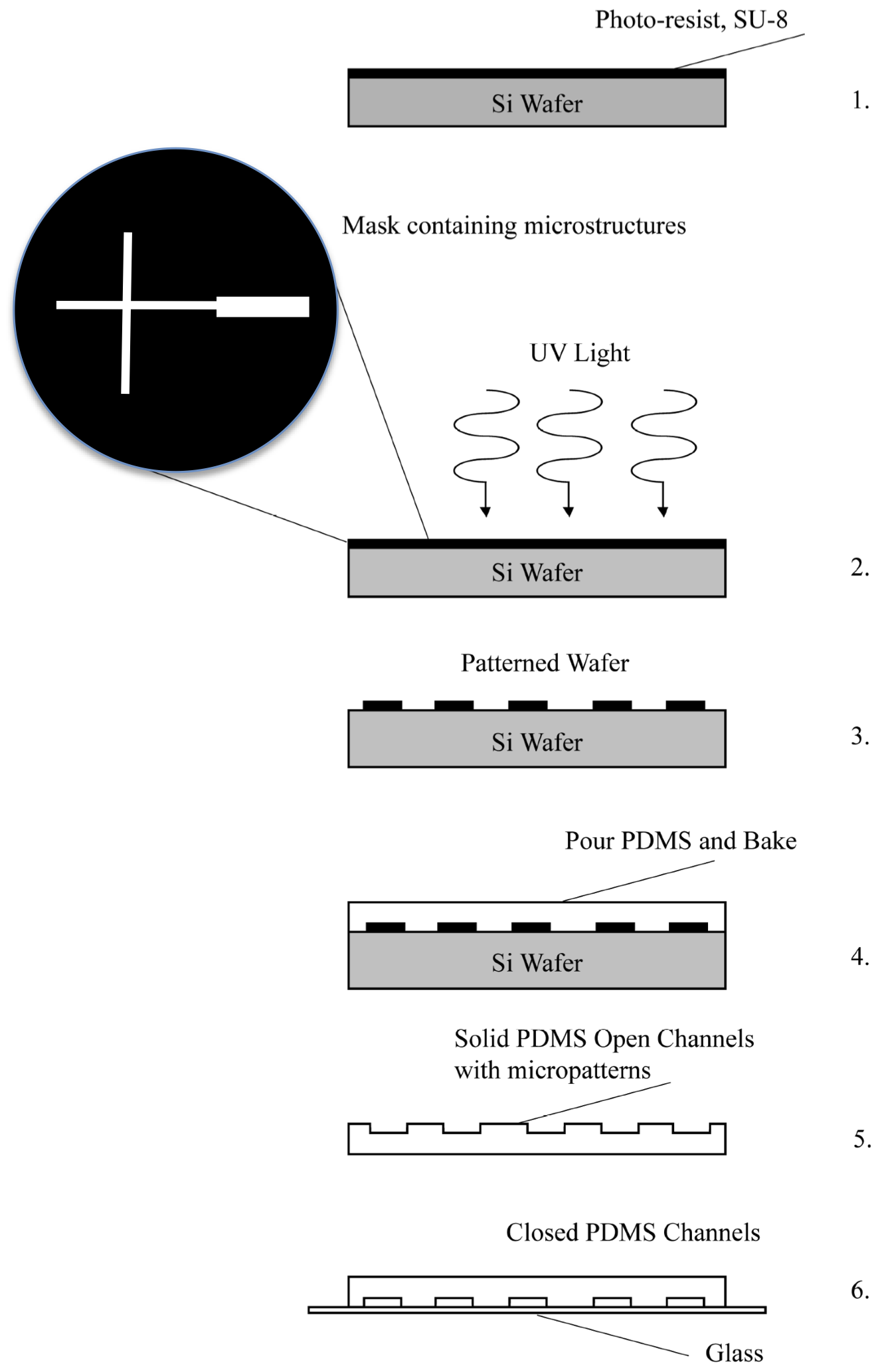

Supplement: Figure S1 — Steps involved in microdevice fabrication. See text in Appendix S1 for details. (TIF) [file pone.0033070.s002.tif]

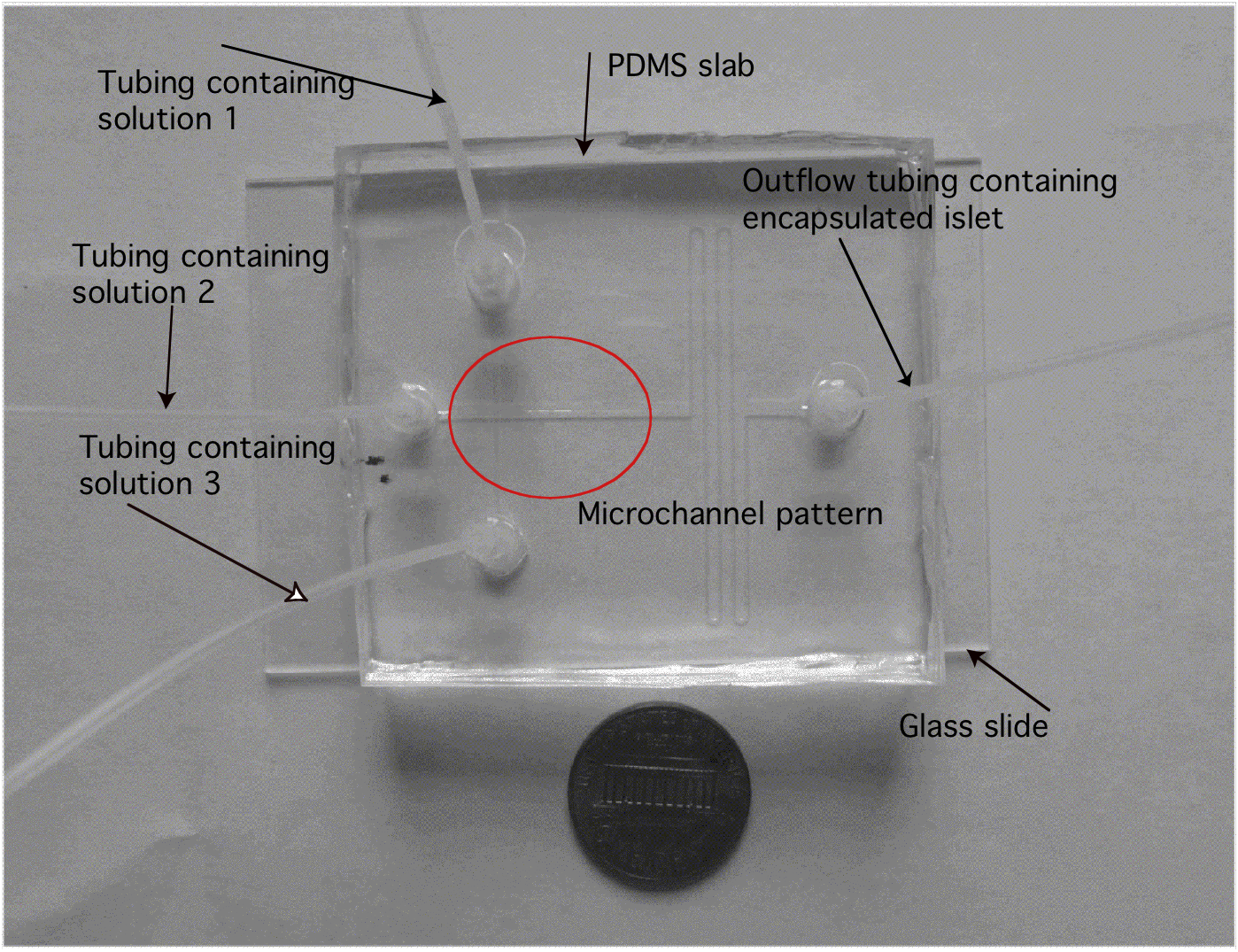

Supplement: Figure S2 — A photo image of the microdevice used for encapsulation of islets. See text in Appendix S1 for details. (TIF) [file pone.0033070.s003.tif]
